# The role of support and other factors in early breastfeeding cessation: an analysis of data from a maternity survey in England

**DOI:** 10.1186/1471-2393-14-88

**Published:** 2014-02-26

**Authors:** Laura L Oakley, Jane Henderson, Maggie Redshaw, Maria A Quigley

**Affiliations:** 1 Policy Research Unit in Maternal Health and Care, National Perinatal Epidemiology Unit, University of Oxford, Old Road Campus, Headington, Oxford OX3 7LF, UK; 2 Department of Non-communicable Disease Epidemiology, London School of Hygiene and Tropical Medicine, Keppel St, London WC1E 7HT, UK

**Keywords:** Breastfeeding, Epidemiology, Public health, Socio-economic

## Abstract

**Background:**

Although the majority of women in England initiate breastfeeding, approximately one third cease breastfeeding by six weeks and many of these women report they would like to have breastfed for longer.

**Methods:**

Data from a survey of women ≥16 years who gave birth to singleton term infants in 2009 in England; questionnaires were completed approximately three months postnatally. Logistic regression was used to investigate the association between postnatal support and other factors, and breastfeeding cessation at 10 days and six weeks. Population attributable fractions (PAFs) were calculated to estimate the relative contribution of breastfeeding support factors to overall breastfeeding cessation at these two time points.

**Results:**

Of the 3840 women who initiated breastfeeding and reported timing of breastfeeding cessation, 13% had stopped by 10 days; and of the 3354 women who were breastfeeding at 10 days, 17% had stopped by six weeks. Socio-demographic factors (maternal age, ethnicity, country of birth, deprivation, education) and antenatal feeding intention were all independently associated with breastfeeding cessation at 10 days and six weeks. Women who did not receive feeding advice or support from a parent or peer support group, voluntary organisation, or breastfeeding clinic were more likely to stop breastfeeding by 10 days. Perceived active support and encouragement from midwives was associated with a lower odds of breastfeeding cessation at both 10 days and six weeks. Estimated PAFs suggest that 34-59% of breastfeeding cessations by 10 days could be avoided if more women in the study population received breastfeeding support.

**Conclusion:**

Although multiple factors influence a mother’s likelihood of continuing breastfeeding, it is clear that socio-demographic factors are strongly associated with breastfeeding continuation. However, there is evidence that breastfeeding support, including that delivered by peer or lay support workers, may have an important role in preventing cessations in the first few weeks.

## Background

The number of mothers in England who initiate breastfeeding has seen a small but steady increase over the last two decades [1] with data from the 2010 UK Infant Feeding Survey (IFS) indicating that 83% of mothers in England now initiate breastfeeding. However, discontinuation rates remain high in the early weeks: nearly one in three mothers who initiate breastfeeding cease by six weeks [1].

The factors which influence whether or not a mother continues breastfeeding may differ from those which affect breastfeeding initiation. A number of studies conducted in the UK and other high-income settings have investigated the association between a range of factors and breastfeeding duration. These studies have identified a strong association between duration and antenatal feeding intention and attitudes, [2-5] and socio-demographic factors including ethnicity, [1,4,6,7] age, [1,2,4,8] maternal education [4,8-10] and socio-economic or area deprivation status [1,7,9,11,12]. In addition, factors relating to maternity care and hospital infant feeding practices are associated with breastfeeding continuation, [2,4,9] though an association with labour and birth factors is less clear [2,6,8,13].

Evidence regarding the effect of breastfeeding support on breastfeeding duration is largely drawn from evaluations of relevant interventions. Although a recently updated Cochrane review suggests that breastfeeding support interventions appear to have a beneficial effect on increasing the number of mothers who breastfeed beyond the immediate postnatal period, [14] results from trials conducted in the UK so far do not provide support for this conclusion. Of the nine UK breastfeeding trials included in a recent narrative review, none reported significant improvement in breastfeeding outcomes, [15] and the five UK-based RCTs included in a systematic review of peer breastfeeding support all failed to demonstrate a significant effect on breastfeeding continuation [16]. Few observational studies have focused on the effect of breastfeeding support, though in a multivariable analysis of 2010 UK IFS data, awareness and/or use of breastfeeding support services was independently associated with breastfeeding continuation at two weeks but not at six weeks postpartum [1].

We used data from a recent national maternity survey to investigate factors associated with breastfeeding cessation in the first six weeks.

## Methods

The objectives of our study were to investigate the factors associated with breastfeeding cessation at 10 days and six weeks, and to assess the relative contribution of breastfeeding support factors to overall breastfeeding cessation at these two time points.

### Study design

We used data from a national survey of women conducted in England in 2010, designed to report on women’s experiences of maternity care, health and wellbeing up to three months postnatally. A random sample of 10,000 women ≥16 years who gave birth in a fortnight period in 2009 was identified by the Office for National Statistics (ONS) using birth registration data. Women whose babies had died were excluded from the sampling frame. Eligible women were sent a questionnaire, letter and information about the study in a range of languages when their babies were approximately three months old. After a series of tailored reminders (a reminder letter after two weeks, a further questionnaire after four weeks), 5,333 women (55.1%) returned a usable questionnaire. The original survey was approved by the Trent Multi-Centre Research Ethics Committee. Further details of the study methodology are published elsewhere [17].

Our analysis was restricted to women who gave birth to singleton infants at term gestation (≥37 weeks gestation). Multiple births and preterm infants were excluded due to small numbers and more complex feeding patterns which may not have been captured in our questionnaire.

### Measures

Breastfeeding cessation was assessed among women who reported initiating breastfeeding, where initiation was defined as the baby receiving any breast milk (hereafter referred to being breastfed), either exclusively or in conjunction with the use of infant formula, in the first few days after birth. Cessation was defined as the point at which the baby no longer received any breast milk. We focused on cessation at two time points: 10 days (among those initiating breastfeeding) and six weeks (among those still breastfeeding at 10 days). These time points were chosen to reflect key points in the postnatal period, with discharge from midwife care usually occurring by day 10 and the routine infant check scheduled to take place at 6–8 weeks.

We included a range of explanatory variables which were grouped as socio-demographic, antenatal and birth, or postnatal support.

The socio-demographic variables included area-based deprivation (Index of Multiple Deprivation quintile – IMD [18]), age of woman, age completed full-time education, cohabitation status, parity, ethnicity and country of birth. Antenatal and birth factors comprised antenatal feeding intention, whether the midwife had discussed infant feeding during antenatal care, gestational age at birth, birthweight, mode of birth, skin to skin after birth, duration of labour, length of postnatal hospital stay, neonatal unit (NNU) admission, and maternal physical health in the first few days. The postnatal support variables included: frequency of postnatal midwife visits, continuity of carer postnatally, baby’s age at last midwife visit, and a number of questions addressing perceived quality of midwife or carer help or advice regarding feeding and the source of any feeding support received (wording for these questions given in Table 1).

**Table 1:** Breastfeeding support questions


Thinking about feeding your baby, did you feel that midwives and other carers gave you:	*Yes, always*
	*Yes, generally*
a) Consistent advice?	*No*
b) Practical help?	*Don’t know*
c) Active support and encouragement?	*Didn’t want this*
	*(Please tick one answer for each of a), b) and c))*
Who helped or advised you with feeding your baby?	*Health professions*
	*Partner/friend/relative*
	*Parent support or peer group*
	*Voluntary organisation*
	*Breastfeeding clinic*
	*I was not given any help or advice*
	*I did not need help or advice*
	*(Please tick all that apply)*
Since your baby was born have the following been available or used by you?	*Was available*
a) A 'baby café’^1^	*Have attended or used*
	*(Please tick one answer or leave blank if service not available)*

^1^'baby cafés’ are a national network of drop-ins providing breastfeeding information and support, led by a health professional or breastfeeding counsellor.

With the exception of IMD which was provided by the ONS, all variables were self-report measures taken from survey data.

### Statistical analysis

Logistic regression modelling was conducted in three stages due to the large number of explanatory variables. Firstly, unadjusted odds ratios (OR) were calculated for the association between each explanatory variable and breastfeeding cessation at both 10 days and 6 weeks. Secondly, these ORs were adjusted for other factors in the same group (socio-demographic, antenatal and birth, postnatal support). Finally, the ORs were adjusted for all explanatory variables together. At each stage explanatory variables were only retained if they were independently associated with breastfeeding cessation, defined as at least one odds ratio at p <0.05 (Wald test). Therefore, variables that did not significantly change the fit of the final model were dropped.

We calculated population attributable fractions (PAFs) for breastfeeding support variables independently associated with breastfeeding cessation at either of the two time points. PAFS were calculated using the formula PAF = p(OR-1)/OR, where 'p’ was the proportion of those who had ceased breastfeeding who reported receiving the specified support and OR was the adjusted odds ratio. Confidence intervals for PAFs were calculated using the formula suggested by Greenland [19].

Univariable analysis was conducted using all available data. For multivariable modelling, analysis was restricted to those observations with complete data on all variables included in the multivariable model. For four variables with 3-15% missing values (country of birth, baby’s age at last MW contact, NNU admission, length of postnatal hospital stay) a further category of 'missing’ was included to enable the variable to be included in modelling without substantial reduction in sample size.

IMD quintile was associated with non-response, [17] hence we weighted the sample for non-response using IMD quintile. All reported frequencies are unweighted and all reported percentages weighted unless otherwise specified. Descriptive analysis is only presented for variables that were statistically significant (p <0.05) in multivariable analysis; additional results are presented as supplementary data (Additional file 1: Table S1).

All statistical analysis was conducted using the survey commands in Stata version 11.

## Results

**Figure 1:**
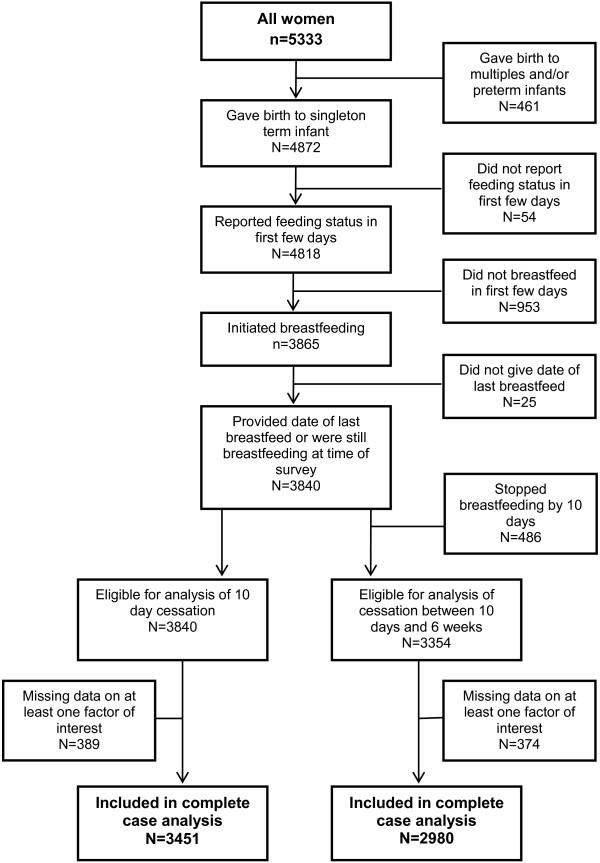
Flowchart of study sample.

**Figure 2:**
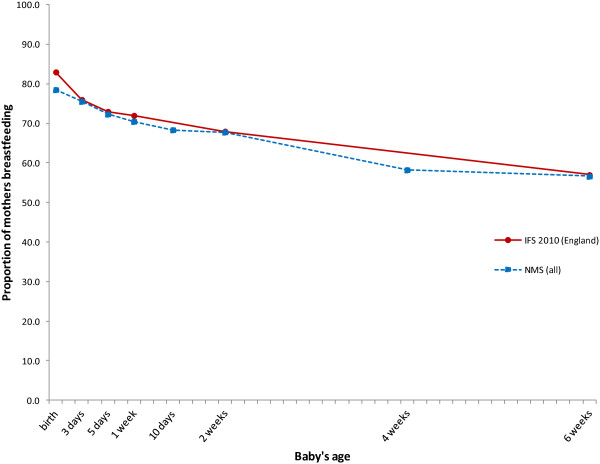
Breastfeeding initiation and duration: survey data and IFS 2010. ^*^Includes mothers who gave birth to preterm infants and/or multiples. ^‡^Data for England only.

Data on initial feeding status were available for 4,818 women (Figure 1). Figure 2 shows the proportion of respondents in the survey who initiated breastfeeding and those still breastfeeding at intervals up to six weeks. The breastfeeding rates are almost identical to those observed in the IFS 2010.

### Breastfeeding cessation at 10 days

Of the 3840 women who initiated breastfeeding and provided information on the timing of any breastfeeding cessation, 13% (n = 486) had stopped breastfeeding by 10 days (Table 2).

**Table 2:** Breastfeeding initiation and cessation by socio-demographic, antenatal and birth characteristics

	**All women**	**Initiated breastfeeding**		**Stopped breastfeeding by 10 days**	**Stopped breastfeeding between 10 days and 6 weeks**
	**n**^**a**^	**n**^**a**^	**(%)**^**b**^	**(%)**^**b,c**^	**(%)**^**b,d**^
**All women**	4818	3865	(79.1)	(13.0)	(16.7)
**Index of Multiple Deprivation**					
Quintile 1 (least deprived)	965	829	(85.9)	(10.0)	(13.6)
Quintile 2	954	801	(84.0)	(11.6)	(14.8)
Quintile 3	1046	859	(82.1)	(13.4)	(18.4)
Quintile 4	896	700	(78.1)	(13.4)	(17.9)
Quintile 5 (most deprived)	956	675	(70.6)	(15.5)	(18.1)
**Age**					
16-19	137	61	(44.8)	(35.6)	(39.9)
20-24	629	384	(60.1)	(24.7)	(26.7)
25-29	1179	943	(79.2)	(13.8)	(18.3)
30-34	1605	1390	(85.9)	(11.2)	(16.3)
35-39	1006	852	(84.3)	(8.2)	(11.3)
≥40	238	215	(89.6)	(8.2)	(11.8)
**Age completed full-time education**					
<17 years	1039	632	(59.0)	(21.4)	(29.6)
17-18 years	1262	946	(74.2)	(19.6)	(23.6)
19+ years	2408	2196	(90.8)	(7.7)	(10.9)
Still in full-time education	65	55	(84.6)	(13.5)	(10.8)
**Ethnicity**					
White	4111	3227	(77.0)	(14.9)	(18.6)
Non-white	658	594	(90.0)	(4.3)	(9.1)
**Country of birth#**					
UK	3668	2848	(75.9)	(16.0)	(19.6)
Outside UK	969	898	(92.7)	(4.3)	(9.1)
**Antenatal feeding intention**					
Breast milk only	3299	3194	(96.6)	(10.9)	(15.0)
Breast and formula milk	618	549	(88.5)	(18.3)	(25.6)
Formula milk only	801	52	(6.4)	(51.4)	(49.8)
Not sure	94	65	(67.4)	(38.3)	(18.8)
**Gestation**					
37-38 weeks	819	616	(74.0)	(13.6)	(18.6)
39-41 weeks	3484	2837	(80.3)	(12.7)	(15.7)
≥42 weeks	515	412	(79.0)	(14.0)	(21.0)
**Type of birth**					
Normal vaginal	3013	2406	(78.5)	(13.4)	(15.6)
Forceps/ventouse	612	500	(80.4)	(13.0)	(17.7)
Planned caesarean	551	418	(75.0)	(9.8)	(20.9)
Unplanned caesarean	584	493	(84.1)	(13.5)	(17.4)
**Duration of labour**					
No labour	522	393	(74.3)	(9.5)	(19.2)
<8 h	1991	1577	(77.8)	(13.6)	(14.6)
8-17 h	1215	992	(80.5)	(13.7)	(18.1)
18+ h	959	804	(83.1)	(12.7)	(18.0)
**Length of hospital stay**^**#**^					
None (home birth)	175	158	(89.4)	(8.7)	(10.1)
<24 h	1612	1260	(76.9)	(11.1)	(15.3)
1-2 days	1564	1265	(79.8)	(15.0)	(18.2)
3-4 days	1120	913	(80.5)	(14.3)	(17.8)
≥5 days	139	107	(76.7)	(7.9)	(19.3)
**Baby admitted to NNU**^**#**^					
Yes	296	216	(72.5)	(8.6)	(18.9)
No	3851	3078	(78.6)	(13.8)	(17.6)

^a^unweighted n, ^b^% weighted for non-response, ^c^of those who initiated breastfeeding and reported timing of any breastfeeding cessation (n = 3840), ^d^of those who were still breastfeeding at 10 days and reported timing of any breastfeeding cessation (n = 3354).

^#^all variables had <3% missing data except for the following variables: country of birth (3% missing), length of hospital stay (4% missing), baby NNU admission (15% missing).

In univariable analysis, cessation at 10 days was significantly associated with all socio-demographic factors, and a number of antenatal and birth factors (antenatal feeding intention, antenatal discussion of infant feeding, labour duration, length of postnatal stay, and NNU admission) and support factors (baby’s age at last contact with midwife, receiving feeding help or advice from a number of specific sources, needing any feeding support) (Tables 2 and 3). In addition, those women who reported that midwives did not give consistent advice regarding feeding, or did not provide active support and encouragement, were more likely to have stopped breastfeeding at 10 days.

**Table 3:** Breastfeeding initiation and cessation by breastfeeding support factors

	**All women**	**Initiated breastfeeding**		**Stopped breastfeeding by 10 days**	**Stopped breastfeeding between 10 days and 6 weeks**
	**n**^**a**^	**n**^**a**^	**(%)**^**b**^	**(%)**^**b,c**^	**(%)**^**b,d**^
**Feeding help/advice from parent support or peer group**					
Yes	504	444	(86.9)	(8.0)	(13.0)
No	4310	3418	(78.2)	(13.6)	(17.2)
**Feeding help/advice from voluntary organisation**					
Yes	264	257	(97.3)	(5.1)	(12.7)
No	4550	3605	(78.1)	(12.7)	(17.1)
**Feeding help/advice from breastfeeding clinic**					
Yes	584	572	(97.8)	(5.0)	(15.4)
No	4230	3290	(76.6)	(13.6)	(17.0)
**Needed help and advice re. feeding**					
Yes	3315	617	(60.3)	(14.2)	(18.1)
No	882	547	(83.4)	(5.5)	(9.4)
**Attended a baby café**					
Yes	283	239	(82.5)	(9.4)	(10.4)
No	874	689	(77.4)	(12.0)	(16.4)
Service not available	3595	2882	(79.2)	(13.6)	(17.4)
**Midwives gave active support and encouragement re. feeding**					
Yes, always	1861	1588	(84.4)	(11.3)	(14.3)
Yes, generally	1927	1603	(82.1)	(13.3)	(18.4)
No	727	489	(65.6)	(19.6)	(21.5)
Don’t know	85	58	(67.4)	(7.0)	(15.8)
Didn’t want this	150	75	(48.7)	(6.6)	(6.0)

^a^unweighted n, ^b^% weighted for non-response, ^c^of those who initiated breastfeeding and reported timing of any breastfeeding cessation (n = 3840), ^d^of those who were still breastfeeding at 10 days and reported timing of any breastfeeding cessation (n = 3354).

In multivariable analysis, the patterns were similar. Most socio-demographic factors were independently associated with cessation at 10 days, although effects were somewhat attenuated by adjustment (Table 4). Area deprivation, younger age, White ethnicity, younger age at completion of full-time education, and UK birth all continued to be associated with increased odds of breastfeeding cessation at 10 days. Older age remained associated with a lower odds of cessation. Antenatal feeding intention remained strongly associated with breastfeeding cessation at 10 days, with those who intended to mixed feed, bottle feed, or with no firm intention all more likely to have ceased breastfeeding by 10 days. Duration of labour (specifically “no labour”) and infant admission to NNU remained associated with a lower odds of cessation, and longer hospital postnatal stay continued to be associated with a higher odds of cessation. In terms of key support variables, women who did not receive feeding advice or support from a parent support/peer group, voluntary organisation or breastfeeding clinic were more likely to stop breastfeeding by 10 days, with little change after adjustment for other factors (Table 5). Compared to women who reported that midwives 'always’ gave active support and encouragement regarding feeding, those who said this support was 'generally’ given or not given at all were more likely to have stopped breastfeeding at 10 days.

**Table 4:** Socio-demographic, antenatal and birth factors associated with breastfeeding cessation in multivariable analysis

	**Breastfeeding cessation by 10 days**				**Breastfeeding cessation between 10 days and 6 weeks**			
	**CRUDE OR (complete case) (n=3451)**		**FINAL ADJUSTED**^**a **^**(complete case) (n=3451)**		**CRUDE OR (complete case) (n=2980)**		**FINAL ADJUSTED**^**b **^**(complete case) (n=2980)**	
	**OR**	**(95% CI)**	**OR**	**(95% CI)**	**OR**	**(95% CI)**	**OR**	**(95% CI)**
**Index of Multiple Deprivation**								
Quintile 1 (least deprived)	1.00	-	1.00	-	1.00	-	1.00	-
Quintile	1.20	(0.86,1.66)	1.07	(0.75,1.54)	1.15	(0.85,1.57)	1.13	(0.82,1.57)
Quintile 3	1.46*	(1.07,2.01)	1.38	(0.99,1.93)	1.49**	(1.11,2.01)	1.57**	(1.15,2.16)
Quintile 4	1.53*	(1.10,2.12)	1.50*	(1.03,2.18)	1.44*	(1.06,1.97)	1.45*	(1.03,2.03)
Quintile 5 (most deprived)	1.75***	(1.26,2.43)	1.84**	(1.27,2.68)	1.55**	(1.13,2.14)	1.79**	(1.25,2.57)
**Age**								
16-19	3.84***	(2.15,6.85)	1.9	(0.97,3.74)	3.32***	(1.69,6.53)	1.84	(0.93,3.63)
20-24	2.39***	(1.75,3.27)	1.51*	(1.04,2.19)	1.73**	(1.24,2.41)	1.24	(0.85,1.79)
25-29	1.22	(0.93,1.59)	1.12	(0.83,1.50)	1.08	(0.84,1.39)	1.02	(0.78,1.33)
30-34	1.00	-	1.00	-	1.00	-	1.00	-
35-39	0.66*	(0.48,0.91)	0.66*	(0.48,0.92)	0.58***	(0.43,0.77)	0.55***	(0.40,0.75)
≥40	0.70	(0.41,1.19)	0.64	(0.37,1.13)	0.61	(0.36,1.01)	0.52*	(0.30,0.89)
**Age completed full-time education**								
<17 years	3.35***	(2.57,4.36)	2.15***	(1.58,2.92)	3.44***	(2.66,4.46)	3.03***	(2.29,4.02)
17-18 years	2.92***	(2.29,3.73)	1.99***	(1.52,2.61)	2.59***	(2.05,3.27)	2.24***	(1.74,2.87)
19+ years	1.00	-	1.00	-	1.00	-	1.00	-
Still in full-time education	1.89	(0.79,4.55)	2.11	(0.86,5.22)	1.21	(0.46,3.22)	1.31	(0.45,3.81)
**Ethnicity**								
White	3.47***	(2.22,5.43)	3.02***	(1.74,5.22)	2.48***	(1.73,3.54)	2.49***	(1.63,3.80)
Non-white	1.00	-	1.00	-	1.00	-	1.00	-
**Country of birth**								
UK	4.29***	(2.89,6.36)	2.91***	(1.85,4.56)	2.58***	(1.92,3.46)	1.71**	(1.63,3.06)
Outside UK	1.00	-	1.00	-	1.00	-	1.00	-
Antenatal feeding intention								
Breast milk only	1.00	-	1.00	-	1.00	-	1.00	-
Breast and formula milk	2.06***	(1.58,2.67)	2.47***	(1.84,3.31)	2.03***	(1.56,2.63)	2.63***	(1.97,3.53)
Formula milk only	8.70***	(4.82,15.71)	6.90***	(2.94,16.20)	6.18***	(2.71,14.10)	4.73**	(1.74,12.87)
Not sure	4.10***	(2.31,7.28)	4.01***	(2.17,7.42)	1.33	(0.57,3.09)	1.09	(0.45,2.63)
**Duration of labour**								
No labour	0.66*	(0.44,0.98)	0.55**	(0.36,0.84)	-	-	-	-
<8 h	1.00	-	1.00	-	-	-	-	-
8-17 h	1.03	(0.79,1.28)	0.97	(0.73,1.29)	-	-	-	-
18+ h	0.98	(0.72,1.21)	0.89	(0.66,1.22)	-	-	-	-
**Length of hospital stay**								
None (home birth)	0.78	(0.43,1.41)	0.88	(0.46,1.71)	-	-	-	-
<24 h	1.00	-	1.00	-	-	-	-	-
1-2 days	1.39*	(1.08,1.78)	1.42*	(1.08,1.88)	-	-	-	-
3-4 days	1.32	(1.00,1.74)	1.78***	(1.29,2.46)	-	-	-	-
≥5 days	0.66	(0.31,1.41)	1.39	(0.59,3.24)	-	-	-	-
**Gestation**								
37-38 weeks	-	-	-	-	1.21	(0.93,1.59)	1.25	(0.93,1.68)
39-41 weeks	-	-	-	-	1.00	-	1.00	-
≥ 42 weeks	-	-	-	-	1.58**	(1.17,2.12)	1.40*	(1.01,1.95)
**Type of birth**								
Normal vaginal	-	-	-	-	1.00	-	1.00	-
Forceps/ventouse	-	-	-	-	1.19	(0.89,1.60)	1.14	(0.84,1.54)
Planned caesarean	-	-	-	-	1.42*	(1.05,1.91)	1.50*	(1.09,2.07)
Unplanned caesarean	-	-	-	-	1.24	(0.91,1.67)	1.30	(0.93,1.81)
**Baby admitted to NNU**								
Yes	0.55*	(0.32,0.93)	0.42**	(0.23,0.78)	-	-	-	-
No	1.00	-	1.00	-	-	-	-	-

^a^Adjusted for IMD, age, age completed FT education, ethnicity, country of birth, AN feeding intention, labour duration, postnatal stay length, NNU admission, source of breastfeeding support, breastfeeding support not needed, MWs gave active support and encouragement.

^b^Adjusted for IMD, age, age completed FT education, ethnicity, country of birth, AN feeding intention, gestation, type of birth, baby café attendance, breastfeeding support not needed, MWs gave active support and encouragement.

*p <0.05 **p <0.005 ***p <0.001.

**Table 5:** Breastfeeding support factors associated with breastfeeding cessation in multivariable analysis

	**Breastfeeding cessation by 10 days**						**Breastfeeding cessation between 10 days and 6 weeks**			
	**CRUDE OR (complete case) (n=3451)**		**FINAL ADJUSTED**^**a **^**(complete case) (n=3451)**				**CRUDE OR (complete case) (n=2980)**		**FINAL ADJUSTED**^**b **^**(complete case) (n=2980)**	
	**OR**	**(95% CI)**	**OR**	**(95% CI)**	**PAF**	**95% CI**	**OR**	**(95% CI)**	**OR**	**(95% CI)**
**Attended a baby café**										
Yes	-	-	-	-	-	-	1.00	-	1.00	-
No	-	-	-	-	-	-	1.68*	(1.02,2.77)	1.47	(0.87,2.50)
Service not available	-	-	-	-	-	-	1.80*	(1.14,2.86)	1.73*	(1.06,2.81)
**Feeding help/advice from parent support or peer group**										
Yes	1.00	-	1.00	-			-	-		
No	1.67**	(1.14,2.46)	1.58*	(1.03,2.41)	34.0%	(2.7-55.2%)	-	-	-	-
**Feeding help/advice from voluntary organisation**										
Yes	1.00	-	1.00	-			-	-	-	-
No	2.72***	(1.84,4.02)	2.65***	(1.76,3.98)	58.1%	(39.1-71.2%)	-	-	-	-
**Needed help and advice re. feeding**										
Yes	2.79***	(1.85,4.20)	2.84***	(1.83,4.40)	-	-	2.36***	(1.66,3.36)	2.32***	(1.58,3.40)
No	1.00	-	1.00	-	-	-	1.00	-	1.00	-
**Midwives gave active support and encouragement re. feeding**										
Yes, always	1.00	-	1.00	-	-	-	1.00	-	1.00	-
Yes, generally	1.22	(0.97,1.54)	1.34*	(1.04,1.72)	-	-	1.34**	(1.08,1.67)	1.58***	(1.25,1.99)
No	1.97***	(1.47,2.64)	2.22***	(1.59,3.11)	-	-	1.73***	(1.28,2.34)	2.01***	(1.45,2.77)
Don’t know	0.54	(0.16,1.83)	0.57	(0.15,2.09)	-	-	0.80	(0.33,1.92)	0.88	(0.39,2.02)
Didn’t want this	0.46	(0.14,1.50)	1.16	(0.37,3.71)	-	-	0.30*	(0.09,0.98)	0.71	(0.23,2.22)

^a^Adjusted for IMD, age, age completed FT education, ethnicity, country of birth, AN feeding intention, labour duration, postnatal stay length, NNU admission, source of breastfeeding support, breastfeeding support not needed, MWs gave active support and encouragement.

^b^Adjusted for IMD, age, age completed FT education, ethnicity, country of birth, AN feeding intention, gestation, type of birth, baby café attendance, breastfeeding support not needed, MWs gave active support and encouragement.

*p <0.05 **p <0.005 ***p <0.001.

### Breastfeeding cessation at six weeks

Of the 3354 women who were breastfeeding at 10 days, 17% (n = 551) had stopped by six weeks (Table 2).

Breastfeeding cessation between 10 days and six weeks was associated with all socio-demographic factors in univariable analysis, with patterns similar to those observed in the analysis of breastfeeding cessation at 10 days (Table 2). Antenatal feeding intention was also strongly associated with cessation at six weeks in univariable analysis, as was gestation, type of birth, duration of labour, and maternal health in the first few days. In terms of support variables, only needing help and advice regarding feeding, attendance at a baby café, and quality of midwifery care regarding feeding (consistency of advice, practical help, active support and encouragement) were associated with a lower odds of breastfeeding cessation between 10 days and six weeks in univariable analysis (Table 3).

Compared to the analysis of breastfeeding cessation by 10 days, a smaller number of factors were independently associated with cessation between 10 days and six weeks (Tables 4 and 5). After adjustment, cessation by six weeks was lowest in older mothers, and highest in women living in the most deprived areas, who had a younger age at completing full-time education, who were White ethnicity, and those born in the UK (Table 4). Antenatal intention to formula feed continued to be significantly associated with increased cessation between 10 days and six weeks, as did higher gestation and planned caesarean. Attendance at a baby café remained in the multivariable model, with a higher odds of cessation for those reporting that this particular service was not available. Women who reported needing help and advice regarding feeding, and those who reported that midwives did not always give active support and encouragement regarding feeding, were more likely to have stopped breastfeeding by six weeks even after adjusting for other factors.

### Population attributable fractions

We calculated PAFs for three variables independently associated with breastfeeding cessation at 10 days: whether women had received feeding help from a parent support or peer group; whether women had received feeding help from a voluntary organisation; and whether women had received feeding help from a breastfeeding clinic (Table 5). The PAFs for these three variables were 34.0%, 58.9% and 58.1% respectively, suggesting that, assuming causality, 34-59% of breastfeeding cessations by 10 days could be avoided if more women in the study population received breastfeeding support.

## Discussion

In our analysis of data from a recent national maternity survey, socio-demographic factors, feeding intention and breastfeeding support were all independently associated with breastfeeding cessation. Apart from breastfeeding support which was only independently associated with cessation by 10 days, there was little difference in the factors associated with cessation at 10 days and six weeks. Although a large number of explanatory variables were included in the univariable analysis and many of these showed associations with breastfeeding cessation, adjustment for other factors attenuated many of these associations.

A strong effect of socio-demographic factors on breastfeeding has been observed in numerous individual level studies [1,2,4,6-11] and also by a recent area level analysis [20]. We found that younger women, women living in more deprived areas, those with lower levels of education, and White women were all more likely to discontinue breastfeeding. It is interesting that not only are these groups less likely to initiate breastfeeding, but that even if they initiate breastfeeding, they are less likely to continue to 10 days, and even if they are breastfeeding at 10 days, they are less likely to continue to six weeks, compared with their counterparts in other socio-demographic groups. Many previous studies investigating breastfeeding duration have included in their sample women who did not initiate breastfeeding, making it difficult to isolate a similar association. However, a small number of studies have focused only on those women who initiated breastfeeding, and these have reported similar socio-demographic trends to those observed here [6,8,9]. The socio-demographic factors considered here are unmodifiable; nevertheless our understanding of these associations is key to the design and targeting of appropriate interventions.

The role of antenatal feeding intention was very strongly associated with breastfeeding in our analysis. In an analysis of data from a maternity survey conducted in England in 2006, a strong effect of antenatal feeding intention on breastfeeding outcomes was observed, [4] with similar associations reported in other studies [2,3,5]. Less than one in five women in this analysis reported intending to formula feed (17%) and few reported being undecided (2%), though these figures may be underestimates given that women may be more likely to retrospectively declare an intention to breastfeed. Feeding intention is known to be associated with socio-demographic characteristics, and is also likely to be strongly correlated with peer infant feeding behaviour and whether the mother herself was breastfed. These two latter factors were identified as predictors of breastfeeding continuation in a recent analysis of 2010 IFS data [1].

We explored the role of breastfeeding support on cessation by 10 days and by six weeks. In our survey women were asked whether they had received feeding help from a number of different sources. Women who received help from a parent or peer support group, voluntary organisation, or a breastfeeding clinic were all less likely to have stopped breastfeeding at 10 days, though there was no significant association between these factors and cessation at six weeks. Our findings confirm that even after adjusting for other factors (including the need for support), the provision of breastfeeding support, particularly from non-health professionals, may have an important role in reducing the number of women who stop breastfeeding in the first few weeks. This finding was supported by substantial PAFs, for example estimating that 59% of breastfeeding cessations are attributable to women who need help with feeding not receiving help and advice from a voluntary organisation. As discussed previously, although descriptive studies have linked breastfeeding support to increases in breastfeeding continuation particularly in the early weeks, [1,21] results from UK trials have generally reported no effect on breastfeeding outcomes, even where peer support has been considered as a stand-alone intervention [16]. In an observational study such as this, it is likely that motivation to seek support partly explains the association between breastfeeding support and breastfeeding cessation. The type of support considered here (from non-health professionals) relies on women proactively seeking support: women with high intention or commitment to breastfeed may be more likely to seek help when problems arise.

A number of birth factors were independently associated with breastfeeding cessation at 10 days and/or 6 weeks. Of the observed associations, one particularly interesting trend was seen for caesareans, in particular planned caesareans which were associated with a higher odds of cessation at 6 weeks. Information was not collected on the reasons for caesarean section (maternal conditions, fetal conditions, or other) so it is difficult to suggest possible explanations for this trend. NNU admission was associated with a lower odds of cessation at 10 days, probably due to the fact many of these mothers may have been given support with feeding whilst their babies received care.

### Strengths and limitations

Overall our study was large, though our response rate was relatively low (55%) and non-responders differed from responders in terms of a number of factors, most notably, IMD quintile [17]. We addressed this bias by weighting the analysis according to IMD quintile. This approach, together with the fact that our figures on breastfeeding prevalence are similar to those reported in the IFS from the same time period, [1] suggests our survey is fairly representative.

In our analysis we had access to data on a wide range of factors potentially associated with breastfeeding. Due to the survey design we were unable to look at exclusivity of breastfeeding beyond the first few days. In addition, information was not collected on previous feeding experiences; previous breastfeeding experience has found to be a predictor of breastfeeding duration in other studies [22] and it is likely to be also associated with the need for breastfeeding support.

The focus of the study was maternity care and the survey was conducted when infants were approximately three months old. Although women were explicitly asked about care from midwives and also from health professionals more generally, we did not ask about support provided by other specific health professionals such as health visitors. Due to the timing of the survey we were unable to investigate longer-term breastfeeding, which is potentially subject to influence from different factors. However, the fact that the survey was carried out early in the postnatal period should have minimised bias in the recall of breastfeeding behaviour and other factors relating to the antenatal and birth period.

Women in our survey were asked to report on their use of breastfeeding support services but we did not seek information on the timing of this. Therefore, misclassification may have occurred in the analysis of 10 day cessation as women who were classified as receiving help from a particular source may have in fact sought help beyond 10 days.

In calculating PAFs, we have assumed causality. However, the relationship between breastfeeding support and cessation is clearly complex, and as discussed earlier, must be considered in the context of other factors such as motivation to seek help and support. Nevertheless, calculating PAFs enables us to consider the *potential maximum* effect of improving breastfeeding support and can be considered a useful addition to the research evidence.

### Implications

In 2011 there were 688,120 births in England [23]. Assuming the initiation rate (79%), and the 10 day (68%) and six week (57%) continuation rates observed in our study, we estimate that approximately 151,000 mothers who started breastfeeding in 2011 stopped by the time their baby was six weeks old. This represents a considerable number of breastfeeding cessations that could potentially be prevented. Of those mothers questioned in the 2010 IFS who had stopped breastfeeding, nearly two-thirds said they would have liked to have breastfed for longer [1].

Multiple factors influence a mother’s likelihood of continuing breastfeeding. These factors clearly need to be addressed by interventions which address the socio-demographic context in which a mother breastfeeds. Despite the lack of effect observed in previous evaluations of breastfeeding interventions in the UK, breastfeeding support remains a central component of addressing low breastfeeding rates, and efforts need to be focused on the most appropriate type of support in terms of timing, intensity and delivery. Our results suggest that breastfeeding support delivered by non-health professionals (peer support, voluntary organisation) and specialist support such as breastfeeding clinics may have an important role in preventing breastfeeding cessations in the first few weeks among women giving birth to term singletons.

## Conclusions

A substantial proportion of women who initiate breastfeeding are no longer breastfeeding at six weeks. Efforts to improve breastfeeding rates in the UK are unlikely to be successful without a strong evidence base regarding the specific factors which are associated with breastfeeding continuation and an attempt to address these factors through tailored interventions. Despite the negative findings from UK trials of breastfeeding support, our results suggest that breastfeeding support may help to prevent breastfeeding cessation in the first few weeks. There is a clear need for further research in order to identify the most appropriate interventions for increasing breastfeeding duration.

## Competing interests

The authors declare that they have no competing interests.

## Authors’ contributions

All authors contributed to the analysis plan, and LO conducted the analysis with guidance from MQ. LO and MQ wrote the initial draft of the manuscript. All authors contributed to critical review and approved the final version of the manuscript.

## Pre-publication history

The pre-publication history for this paper can be accessed here:

http://www.biomedcentral.com/1471-2393/14/88/prepub

## Supplementary Material

Additional file 1: Table S1 Breastfeeding initiation and cessation by additional explanatory factors not associated with breastfeeding cessation in multivariable analysis. Click here for file
