# Supplementary material for: The role of support and other factors in early breastfeeding cessation: an analysis of data from a maternity survey in England
Source: BMC Pregnancy Childbirth. 2014 Feb 26;14:88. doi: 10.1186/1471-2393-14-88 (PMC3939405; doi:10.1186/1471-2393-14-88)
Supplement: Additional file 1: Table S1 — Breastfeeding initiation and cessation by additional explanatory factors not associated with breastfeeding cessation in multivariable analysis. [file 1471-2393-14-88-S1.doc]

**Table S1. Breastfeeding initiation and cessation by additional explanatory factors not associated with breastfeeding cessation in multivariable analysis**

^a^unweighted n, ^b^% weighted for non-response, ^c^of those who initiated breastfeeding and reported timing of any breastfeeding cessation (n=3840), ^d^of those who were still breastfeeding at 10 days and reported timing of any breastfeeding cessation (n=3354)

# all variables had <3% missing data except for the following variables: baby’s age at last MW contact (7% missing), birthweight (4% missing)
